# Interaction Effects of Temperature and Ozone on Lung Function and Markers of Systemic Inflammation, Coagulation, and Fibrinolysis: A Crossover Study of Healthy Young Volunteers

**DOI:** 10.1289/ehp.1307986

**Published:** 2014-12-16

**Authors:** Juliette J. Kahle, Lucas M. Neas, Robert B. Devlin, Martin W. Case, Michael T. Schmitt, Michael C. Madden, David Diaz-Sanchez

**Affiliations:** Environmental Public Health Division, National Health and Environmental Effects Research Laboratory, U.S. Environmental Protection Agency, Research Triangle Park, North Carolina, USA

## Abstract

**Background:**

Trends in climate suggest that extreme weather events such as heat waves will become more common. High levels of the gaseous pollutant ozone are associated with elevated temperatures. Ozone has been associated with respiratory diseases as well as cardiovascular morbidity and mortality and can reduce lung function and alter systemic markers of fibrinolysis. The interaction between ozone and temperature is unclear.

**Methods:**

Sixteen healthy volunteers were exposed in a randomized crossover study to 0.3 ppm ozone and clean air for 2 hr at moderate (22°C) temperature and again at an elevated temperature (32.5°C). In each case lung function was performed and blood taken before and immediately after exposure and the next morning.

**Results:**

Ozone exposure at 22°C resulted in a decrease in markers of fibrinolysis the next day. There was a 51.8% net decrease in PAI-1 (plasminogen activator inhibitor-1), a 12.1% net decrease in plasminogen, and a 17.8% net increase in D-dimer. These significantly differed from the response at 32.5°C, where there was a 44.9% (*p* = 0.002) and a 27.9% (*p* = 0.001) increase in PAI-1 and plasminogen, respectively, and a 12.5% (*p* = 0.042) decrease in D-dimer. In contrast, decrements in lung function following ozone exposure were comparable at both moderate and elevated temperatures (forced expiratory volume in 1 sec, –12.4% vs. –7.5%, *p* > 0.05). No changes in systemic markers of inflammation were observed for either temperature.

**Conclusion:**

Ozone-induced systemic but not respiratory effects varied according to temperature. Our study suggests that at moderate temperature ozone may activate the fibrinolytic pathway, while at elevated temperature ozone may impair it. These findings provide a biological basis for the interaction between temperature and ozone on mortality observed in some epidemiologic studies.

**Citation:**

Kahle JJ, Neas LM, Devlin RB, Case MW, Schmitt MT, Madden MC, Diaz-Sanchez D. 2015. Interaction effects of temperature and ozone on lung function and markers of systemic inflammation, coagulation, and fibrinolysis: a crossover study of healthy young volunteers. Environ Health Perspect 123:310–316; http://dx.doi.org/10.1289/ehp.1307986

## Introduction

Over the past decades, air quality in the United States has improved significantly. Even so, millions of people in the United States still live in counties that do not meet air quality standards for one or more pollutants (American Lung Association 2013). Ground-level ozone, one of the main culprits, has been shown to be associated with adverse pulmonary effects such as worsening or promoting asthma [U.S. Environmental Protection Agency (EPA) 2013]. More recently, associations between long-term ozone exposure and cardiovascular morbidity and mortality have been reported (Chen et al. 2007; Forbes et al. 2009).

Ozone is more likely to reach unhealthy levels in urban environments when the weather is dry and hot (U.S. EPA 2013). Over the last century, global temperatures have risen and are projected to continue to rise and cause changes in weather and climate. As a consequence, more frequent and severe heat waves have been forecast. However, despite almost 30 years of research into the effects of ozone, until recently there have been very few studies of the interaction between ozone and temperature on human health. These recent epidemiologic studies have suggested that ozone may modify the associations between temperature and cardiovascular mortality (Dear et al. 2005; Filleul et al. 2006; Pattenden et al. 2010; Ren et al. 2008, 2009). To understand the public health impact of climate change, we need to understand how physiological responses to elevated temperature are affected by the additional stressor of air pollution.

Controlled human exposure studies of healthy and asthmatic individuals have been critical in demonstrating that ozone can cause decrements in lung function (Adams 2003, 2006; Folinsbee et al. 1988; Hazucha 1987; Horstman et al. 1990; McDonnell et al. 1991; Silverman et al. 1976) and lung inflammation (Devlin et al. 1991, 1996; Schelegle et al. 1991). However, nearly all of these studies have been conducted at moderate temperatures (21–23°C). Those studies performed at higher temperatures have centered mainly on impairment of exercise performance in athletes and have shown inconsistent effects on lung function (Folinsbee et al. 1977; Foster et al. 2000; Gibbons and Adams 1984; Gong et al. 1986). None of these studies examined nonrespiratory outcomes. Yet it is becoming clear that both ozone and temperature may have systemic and cardiac effects. The biological mechanisms by which ozone affects cardiovascular risk are not clear because ozone reacts rapidly with respiratory tissues and is not thought to be absorbed or transported to extrapulmonary sites. One commonly proposed mechanism is the promotion of systemic inflammation, endothelial dysfunction, and alteration of coagulation pathways (Brook et al. 2004). In support of this, in a recent study we demonstrated that controlled exposure to ozone can cause an increase in vascular markers of inflammation and alter markers of fibrinolysis (the physiological breakdown of blood clots) (Devlin et al. 2012). Although other mechanisms such as neurogenic modulation may be involved, here we chose to focus on these systemic pathways because high heat has also been shown to be associated with coagulation activation (Bouchama et al. 2012; Meyer et al. 2013).

Here we report on the first, to our knowledge, controlled human exposure study to test vascular and systemic responses to ozone at an elevated temperature. We show that although changes in lung function following ozone exposure are similar at both moderate and high temperatures, there are significant differences in markers of vascular responses to exposure according to temperature, demonstrating an interaction between heat and ozone on key agents of fibrinolysis.

## Materials and Methods

*Study population*. Sixteen (14 males, 2 females) healthy volunteers 21–36 years of age participated in the study. A detailed medical history and physical examination was performed on all participants to ensure that they had no cardiac, allergic, or pulmonary disease. Baseline values for all participants for forced expiratory volume in 1 sec (FEV_1_) and forced vital capacity (FVC) were at least 80% predicted for sex, height, and age. All participants were lifetime nonsmokers. Body mass index (BMI) ranged from 16.6 to 29.8. For a summary of the study population at baseline, see Table 1. All aspects of the study including protocols detailing procedures, recruitment materials, and consent forms were reviewed and approved by the Biomedical Institutional Review Board at the University of North Carolina at Chapel Hill School of Medicine and the U.S. EPA. All study participants were informed of the procedures and potential risks and gave written consent for their participation.

**Table 1:** Subject anthropometric and baseline analyte data.

Characteristic	22°C	32.5°C
No. of subjects (male/female)	16 (14/2)	16 (14/2)
Age [years (range)]	27 (20–35)	27 (21–36)
Race/ethnicity (C/H/AA) (*n*)	12/1/3	12/1/3
Height [cm (range)]	178 (154–191)	178 (155–191)
Weight [kg (range)]	79.7 (43.6–97.3)	79.8 (46.7–97.3)
BSA [m^2^ (range)]	1.97 (1.38–2.19)	1.97 (1.42–2.19)
BMI [kg/m^2^ (range)]	25.02 (17.19–32.16)	24.98 (16.57–29.79)
PAI-1 [ng/mL (mean ± SD)]	6.96 ± 16.79	13.58 ± 35.62
D-dimer [ng/mL (mean ± SD)]	111.7 ± 114.3	132.1 ± 51.1
tPA [ng/mL (mean ± SD)]	2.71 ± 2.94	2.26 ± 1.66
vWF [% (mean ± SD)]	94.4 ± 32.9	131.7 ± 42.3
Plasminogen [% (mean ± SD)]	166.8 ± 52.8	119.5 ± 30.0
IL-1B [pg/mL (mean ± SD)]	0.41 ± 1.05	0.32 ± 0.24
IL-6 [pg/mL (mean ± SD)]	1.08 ± 0.63	1.22 ± 0.60
IL-8 [pg/mL (mean ± SD)]	2.71 ± 1.30	2.74 ± 0.86
TNFα [pg/mL (mean ± SD)]	4.39 ± 21.15	4.02 ± 0.96
CRP [ng/mL (mean ± SD)]	680.12 ± 708.87	842.90 ± 1135.75
Abbreviations: AA, African American; BSA, body surface area; C, Caucasian; H, Hispanic.		

*Study design*. Each individual underwent two identical randomized crossover studies, one at moderate temperature (22°C) and one at elevated temperature (32.5°C). In each pair of exposures the participant was blinded to the exposure (clean air or ozone), but we could not blind participants to the temperature. In each case the participant was exposed to 0.3 ppm ozone or clean air for 2 hr; the exposures were separated by at least 1 week (range, 7–441 days) within each temperature exposure; and the elevated temperature exposure began a minimum of 8 weeks (range, 57–484 days) after completion of the moderate temperature exposure pair.

Thus each participant received a total of two clean air and two ozone exposures. At each temperature, the order of the clean air and ozone exposures was randomized and counterbalanced so that half of the test participants first received ozone and the rest first received clean air. To improve safety monitoring, the temperature at which they received exposures was not randomized. Participants had to complete both exposures at moderate temperature with no adverse effects before they were permitted to continue the study and repeat the procedures at the higher temperature. All participants completed the entire study, and no adverse events were reported. All exposures began at the same time in the morning to avoid having circadian fluctuations as a confounding effect.

Exposures were performed as previously described (Devlin et al. 2012; Hernandez et al. 2010). Briefly, all exposures were conducted at the U.S. EPA Human Studies Facility on the campus of the University of North Carolina, Chapel Hill. The chamber was maintained at 40% relative humidity for all exposures. Ozone was generated by a silent electric discharge method (model 502; Meckenheim, Bonn, Germany) and did not deviate beyond 0.001 ppm of the target concentration (0.3 ppm). Clean air was filtered conditioned air that had no detectable levels of ozone, particles, or other pollutants. Temperature was maintained within 0.3°C of the target temperature. Because the aim of the study was to represent pollutant exposure during a heat wave, we performed exposures only when the previous day’s mean ambient temperature was < 24°C in Chapel Hill.

*Study protocol and measurements*. Each participant underwent an exposure to either clean air or ozone (0.3 ppm) for 2 hr while performing alternating periods of 15-min intermittent exercise and 15 min of rest. During the exercise, minute ventilation was measured, and bike wattage was adjusted to maintain a constant rate of VE_min_ = 25 L/min/body surface area (BSA). Spirometry as well as safety end points (symptom questionnaire, breath sounds, and vital signs) were assessed before and immediately after each exposure, and again the next morning, 24 hr after the exposure started; there were no significant changes in safety end points between the different exposures. Spirometry (FVC, FEV_1_) was performed according to American Thoracic Society guidelines (Miller et al. 2005) using a Sensormedics Vmax 220 instrument and software (Sensormedics Corp., Yorba Linda, CA) as previously reported (Kim et al. 2011).

Venous blood was drawn before the exposure and again 1 hr after the exposure ended (post). The participant returned the following day for the final blood draw 24 hr after the exposure started (follow-up). Cytokines and coagulation and fibrinolytic blood markers were measured using the MesoScale Discovery, multiplex platform (Gaithersburg, MD). The cytokines interleukin (IL)-1β, IL-6, IL-8, and tumor necrosis factor (TNF) α were measured using the Human Proinflammatory Panel II kit. C-reactive protein (CRP) was measured using the Human CRP kit and all other assays [D-dimer, tPA (tissue plasminogen activator), vWF (Von Willebrand factor), and plasminogen] were measured using established Multiarray plates per manufacturers’ instructions. A differential blood count and blood lipid panel were performed by LabCorp (Burlington, NC).

*Statistical analysis*. To account for intra- and interindividual heterogeneity and thereby minimize confounder effects, end points measured at 1 hr (post) and 24 hr (follow-up) following exposure were divided by preexposure values and expressed as percent of the baseline (preexposure) (Devlin et al. 2012). A *p*-value of ≤ 0.05 was considered significant. Random mixed-effects models with random participant intercepts for each of the 16 participants were parameterized to estimate directly the contrasts between clean air and ozone within temperature strata. A “day of study” (DOS) factor was added to disentangle temperature and period because the temperature was not randomized. The DOS was calculated for each participant for each exposure with clean air at 22°C set as day 0. Because the order within each exposure pair was randomized, the range of DOS for ozone at 22°C was from 63 days before clean air to 441 days after clean air (–63, 441). The shortest time to complete all four exposures was 93 days and the longest was 526 days. The principal outcome variable was the percent change in outcome of interest between the sample obtained before exposure and the sample obtained 1 or 24 hr later. The random mixed-effects models were fit using R i386 version 3.0.1 with the lme4 package version 0.999999–2 (http://www.r-project.org/foundation/). The CHALLENGE.R code and data used to generate the tables and figures are available on request. We used the following equations for our mixed-effects models:

*E*(*Y*) = α + β_1_*T* + β_2_*AP* + β_3_(*T* × *AP*) + β_4_*DOS* + (1|*Participant*), [1]

where β_1_ is the main effect of temperature; β_2_ is the main effect of ozone versus clean air; β_3_ is the interaction of ozone and temperature; β_4_ is the linear effect of DOS; and (1|*Participant*) is a random participant intercept. The *p*-value for the interaction was calculated from the *t* distribution with *n* – 1 degrees of freedom.

*E*(*Y*) = α + β_1_*T* + β*´*_2_[(1 – *T*) × *AP*] + β*´*_3_(*T* × *AP*) + β_4_*DOS* + (1|*Participant*). [2]

Here, β_1_ is the main effect of temperature; β*´*_2_ is the stratum-specific effect of ozone when *T* = 0; β*´*_3_ is the stratum-specific effect of ozone when *T* = 1; β_4_ is the linear effect of DOS; and (1|*Participant*) is a random participant intercept.

## Results

**Figure 1:**
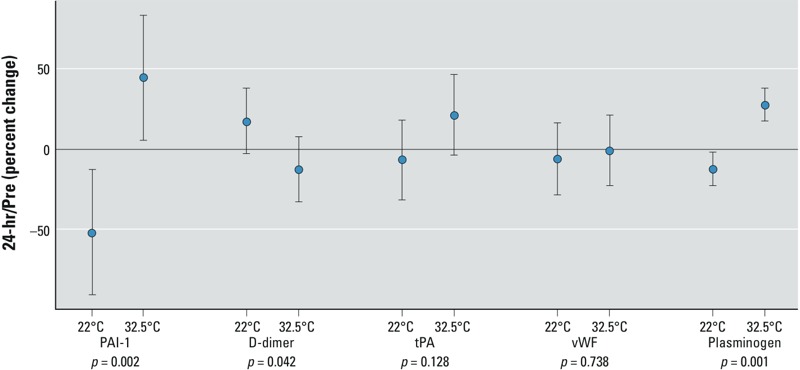
Markers of coagulation after 2 hr ozone exposure at 0.3 ppm, with effects for moderate (22°C) and high (32.5°C) temperatures; the effect of ozone on coagulation markers on the late time point is shown. The point estimate of the mean (effect size) and 95% CIs for 16 subjects are shown. *p*-Values are for ozone–temperature interaction.

*Effect of ozone at different temperatures on vascular markers of coagulation and fibrinolysis*. The effect of ozone on vascular markers of fibrinolysis varied according to the temperature. Table 2 shows the 24-hr/preexposure changes in coagulation and fibrinolysis markers for clean air and ozone exposure under the two temperature conditions. The effect size and confidence intervals (CIs) of ozone at each temperature for the follow-up (24-hr) values are shown in Figure 1. Consistent with our previous report (Devlin et al. 2012), ozone at moderate (22°C) temperature caused a significant decrease in values of both PAI-1 (plasminogen activator inhibitor-1) (–51.8%; 95% CI: –90.8, –12.7) and plasminogen (–12.1%; 95% CI: –22.3, –1.8) 24 hr after exposure compared with preexposure. In contrast, at the elevated (32.5°C) temperature, there was a statistically significant rise in both of these markers (PAI-1, 44.9% increase; 95% CI: 5.9, 83.9, *p* = 0.002; plasminogen, 27.9% increase; 95% CI: 17.1, 38.2, *p* = 0.001). Correspondingly there was a highly significant interaction between temperature and ozone for both PAI-1 (*p* = 0.002) and plasminogen (*p* = 0.001) in our model. There was a 12.5% decrease (95% CI: –32.7, 7.6) in 24-hr/preexposure values for the fibrin degradation product D-dimer under elevated temperature compared with a 17.8% increase (95% CI: –2.4, 38.0) under moderate temperature. Although neither of these changes alone reached statistical significance, when taken together there was a significant temperature–ozone interaction (*p* = 0.042). No significant changes in 24-hr/preexposure values at either temperature or any temperature–ozone interactions were observed for either tPA or vWF.

**Table 2:** Changes in coagulation and fibrinolytic blood markers.

Marker	22°C 24-hr/preexposure^*a*^		32.5°C 24-hr/preexposure^*a*^	
	Air	Ozone	Air	Ozone
PAI-1	34.5% (7.0, 62.1)	–18.2% (–45.8, 9.3)	3.0% (–24.6, 30.5)	47.8% (20.3, 75.4)
D-dimer	–10.7% (–25.2, 3.9)	7.1% (–7.4, 21.6)	2.6% (–11.9, 17.1)	–10.0% (–24.5, 4.6)
tPA	9.8% (–7.9, 27.4)	3.0% (–14.6, 20.7)	–2.6% (–20.3, 15.0)	18.7% (1.0, 36.3)
vWF	11.3% (–4.5, 27.0)	4.6% (–11.7, 20.8)	10.1% (–5.7, 25.8)	9.5% (–6.3, 25.2)
Plasminogen	2.8% (–5.4, 11.0)	–9.5% (–17.7, –1.3)	8.3% (0.1, 16.5)	36.3% (28.1, 44.5)
^***a***^Mean values (95% CIs) at follow-up visit 24-hr after exposure compared with preexposure values for 16 subjects.				

**Figure 2:**
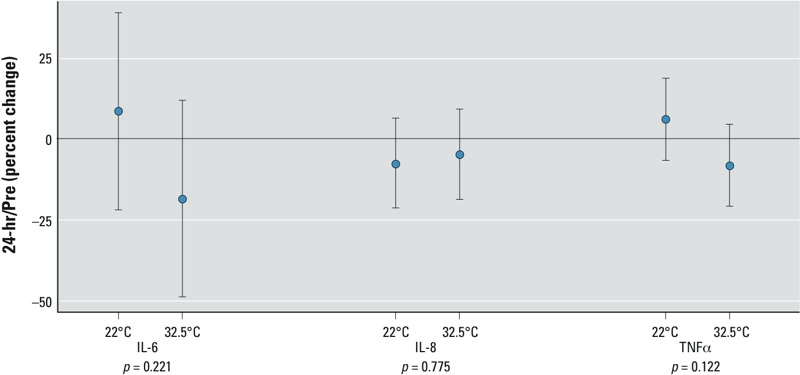
Markers of inflammation after 2 hr ozone exposure at 0.3 ppm, with effects for moderate (22°C) and high (32.5°C) temperatures; the effect of ozone on systemic inflammation markers on the late time point is shown. The mean (effect size) and 95% CIs for 16 subjects are shown. *p*-Values are for ozone–temperature interaction.

*Effect of ozone on vascular markers of inflammation*. Several epidemiologic studies have suggested that ozone can alter vascular inflammation (Bind et al. 2012; Chuang et al. 2007; Thompson et al. 2010). Accordingly, we measured blood concentrations of several factors identified as markers of systemic inflammation. Table 3 shows the 24-hr/preexposure changes in these markers for clean air and ozone exposure under the two temperature conditions. No significant changes in 24-hr/preexposure values at either temperature or any temperature–ozone interactions were observed for IL-6, IL-8, and TNFα (Figure 2). We had previously reported an increase in IL-1β after ozone at moderate temperature at this timepoint (Devlin et al. 2012). Here with a smaller sample size, we could not find a similar rise because of the very large heterogeneity in responses and the corresponding large CIs at either moderate (95% CI: –100.8%, 685.0%) or elevated (95% CI: –438.2%, 346.5%) temperatures. Similarly, for CRP there were very large CIs at moderate (95% CI: –97.5%, 205.1%) and high (95% CI: –188.4%, 113.8%) temperatures, which were driven predominantly by three individuals. For the 1-hr-post time point, there was a trend for all the inflammatory markers to be elevated following ozone exposure at moderate temperature, but none reached statistical significance (data not shown). There was no significant ozone–temperature interaction observed for any of the end points at this time point.

**Table 3:** Changes in systemic inflammation markers.

Marker	22°C 24-hr/preexposure^*a*^		32.5°C 24-hr/preexposure^*a*^	
	Air	Ozone	Air	Ozone
IL-1β	52.6% (–222.5, 327.8)	343.66% (68.5, 618.8)	43.1% (–232.0, 318.3)	–2.8% (–277.9, 272.4)
IL-6	21.6% (–2.4, 45.2)	28.7% (4.7, 52.6)	7.4% (–16.5, 31.4)	–11.0% (–35.0, 13.0)
IL-8	–2.3% (–12.7, 8.1)	–10.7% (–21.1, –0.3)	0.1% (–10.3, 10.5)	–4.6% (–14.9, 5.9)
TNFα	3.2% (–5.9, 12.4)	8.8% (–0.4, 17.9)	4.4% (–4.7, 13.6)	–3.7% (–12.8, 5.5)
CRP	56.2% (–50.5, 162.8)	106.5% (–0.1, 213.2)	94.0% (–12.6, 200.7)	56.5% (–50.1, 163.2)
^***a***^Mean values (95% CIs) at follow-up visit 24-hr after exposure compared with preexposure values for 16 subjects.				

**Figure 3:**
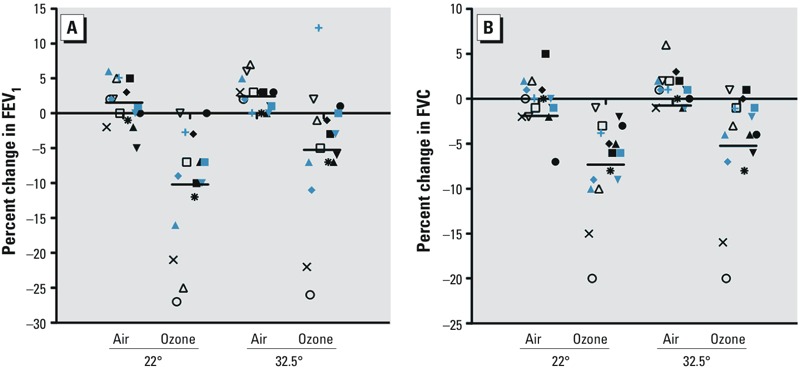
Lung function after 2 hr ozone exposure at 0.3 ppm. Percent changes in (*A*) FEV1 and (*B*) FVC after 2 hr exposure to clean air and 0.3 ppm ozone at either moderate (22°C) or high (32.5°C) temperature. Each participant is denoted by the same shape for each arm. The black horizontal bars are the means for each condition.

*Effect of ozone on lung function*. Ozone-induced lung function decrements immediately following exposure have been reported consistently in multiple studies. Here, we confirmed these previous findings and observed a 12.4% decrease (95% CI: –17.0, –6.1) at moderate temperature in post/preexposure FEV_1_ values following ozone compared with that seen in the same individuals following clean air exposure (Figure 3A). At elevated temperature we saw a 7.5% decrease (95% CI: –13.1, –2.3) in post/preexposure FEV_1_ values. However, there was no statistical difference between the two temperatures and no ozone–temperature interaction (*p* > 0.05). Similarly, temperature did not modulate the effect of ozone on FVC (Figure 3B). There was a 7.5% decrease (95% CI: –10.4, –3.5) in post/preexposure FVC values following ozone compared with clean air at moderate temperature and a 5.9% decrease (95% CI: –9.3, –2.4) at elevated temperature. Again, no ozone–temperature interaction (*p* > 0.05) was observed. These findings agree with a study of well-trained endurance athletes who completed a time-trial 8-km run in moderate and high temperatures with and without ozone and did not have changes in lung function between the conditions (Gomes et al. 2010).

## Discussion

The relationship between temperature and cardiac disease is unclear. Here, we chose to perform a controlled human exposure study because it can safely provide direct evidence of mechanistically relevant biological changes without the confounders inherent in epidemiologic studies (Buckley et al. 2014; Rom et al. 2013). By exposing the same person to both clean air and ozone at a moderate and a high temperature we provide the first direct evidence of an interaction between ozone and temperature that results in changes in the fibrinolysis pathway. We show the ability of ozone at an elevated temperature to affect factors that are involved in preventing blood clots growing and causing thrombosis.

A sudden increase in ambient temperatures has been associated with increased mortality in multiple epidemiology studies (reviewed by Basu 2009), particularly in cardiovascular disease and respiratory mortality. For example Basu and Malig (2011) found a 4% (95% CI: 3.4%, 5.2%) increase in cardiovascular mortality associated with a 5.6% increase in mean daily apparent temperature in California. This mortality can occur even at moderate temperatures; it appears it is not the absolute temperature but the degree of change that is important (Näyhä 2007). Similarly, multiple studies have shown associations between ozone and daily mortality (Bell et al. 2004; Ito et al. 2005), and several have used multicity comparisons and established positive associations between short-term ozone exposure and cardiovascular mortality (Katsouyanni et al. 2009; Stafoggia et al. 2010; Zanobetti and Schwartz 2008). Furthermore, several studies of population-based data have also demonstrated a correlation between ozone and cardiovascular morbidity and mortality, including ischemic stroke and out-of-hospital cardiac arrest (Henrotin et al. 2007; Raza et al. 2014; Rosenthal et al. 2013).

Epidemiology studies assessing the ozone–temperature–cardiac relationship have generally proven problematic because high-ozone days normally occur during hot weather. In addition, the strength of the correlation of ozone with other pollutants such as particulate matter is seasonal and may be temperature dependent. Traditional methods have not proven suitable to discriminate between the effects of ozone and temperature, let alone their interaction. Those that have studied the relationship have produced conflicting results, often because they have treated ozone as a confounder (Reid et al. 2012). When ozone and temperature were treated as co-exposures, researchers have sometimes shown a negative association between temperature and ozone–mortality (Katsouyanni et al. 2009) that may be ascribed to the increased use of air conditioning during hot weather. Recent studies, however, have used novel approaches to determine whether there are joint or interactive effects of ozone and temperature. Ren et al. (2009) examined whether ozone modified the associations between temperature and cardiovascular mortality in 95 large communities in the United States during the summers between 1987 and 2000. They found that a 10°C increase in temperature on the same day was associated with an increase in mortality by 1.17% and 8.31% for the lowest and highest levels of ozone concentrations in all communities, respectively (Ren et al. 2009). Reid et al. (2012) have applied directed acyclic graphs and concluded that ozone is a causal intermediate that is affected by temperature and that can also affect mortality, rather than a confounder. Burkart et al. (2013), using various Posisson regression models, showed interactive effects between air pollution and temperature in Berlin, Germany, and Lisbon, Portugal, that were positively associated with increased excess mortality.

The physiological mechanisms by which either heat or ozone affect cardiovascular events are still not well elucidated. Although multiple mechanisms may be involved, including localized lung inflammation without systemic inflammation or neurogenic modulation, in this study we focused on the role of systemic inflammation and a subsequent disruption of fibrinolysis pathways because both heat and ozone can alter coagulation (Bouchama et al. 2012; Meyer et al. 2013). Endothelium disruption and increased risk of thrombosis has been suggested as a mechanism by which high temperature increases cardiovascular mortality (Näyhä 2005). Heat stress highly activates fibrinolysis and increases levels of D-dimer and decreases levels of plasminogen (Bouchama and Knochel 2002). In this study, although participants were closely monitored so that heat stress did not occur, we believe a similar process is occurring. Consistent with this, our previous complementary time-series panel study looked at the effect of temperature decreases on these markers (Schäuble et al. 2012). We observed that a drop in temperature of 5°C was associated with a significant increase in fibrinogen and PAI-1. Ozone has also been suggested to cause systemic inflammation secondary to lung injury. Multiple epidemiologic studies have examined associations between ozone concentrations and markers of coagulation and inflammation (Chuang et al. 2007; Liao et al. 2005; Rudez et al. 2009). The results from these studies have been inconsistent and have depended on the health of the study population, their ages, and the approach of the authors in disentangling ozone effects from that of other pollutants. Using a controlled human study design to minimize these confounders, we have previously reported that ozone exposure can cause an increase in systemic inflammation, as evidenced by IL-1β and CRP (Devlin et al. 2012). Here, although both markers were also elevated, the very large heterogeneity in responses in this study did not permit us to detect any significant changes with our smaller number; however, an increase in the proinflammatory cytokine IL-6 postexposure was observed.

Although we cannot rule out the possibility that our observations are a result of chance given our multiple comparisions, in both the previous study and here we observed changes consistent with modulation of the fibrinolytic pathway 24 hr after ozone exposure. Plasminogen is the substrate for plasmin, which breaks down fibrin clots. Plasminogen is converted to plasmin by tPA, which is in turn inhibited by PAI-1. A consequence of this process is the formation of fibrin degradation products, of which D-dimer is the most studied. At moderate temperature, the decrease in PAI-1, the parallel decrease in plasminogen and the increase in D-dimer observed are all consistent with activation of fibrinolysis. However, at elevated temperature exactly the opposite response was observed: an increase in PAI-1 and a concomitant increase in plasminogen and decrease in D-dimer. Although we did not observe significant changes in tPA, it is normally released into the blood very slowly by damaged endothelium over several days, so 24 hr may be too early to detect differences. In normal conditions, excessive fibrinolysis is prevented by PAI-1 which is an acute phase reactant protein. We hypothesize that the effect of the combined stimulation of the fibrinolytic pathway by both ozone and heat results in the triggering of this compensatory mechanism and the overproduction of PAI-1. The fibrinolytic pathway is complex and the clinical consequence of the disruption is hard to predict. While this may have no symptomatic importance in healthy individuals, in susceptible populations this reduction in the ability to dissolve clots may result in clinically significant outcomes.

The ability of ozone to impair lung function has been noted in numerous studies. Here we confirmed those findings but saw no difference in lung function changes between moderate and high temperatures. Earlier studies have reported different results depending on the concentration of ozone, level of exercise, the humidity and the population studied. Our study is in agreement with Foster et al. (2000), who found no difference in FEV_1_ responses to 0.5 ppm ozone between 25 and 31°C. Folinsbee et al. (1977), also at 0.5 ppm ozone, examined pulmonary function at different temperatures and found that the greatest decrease in FVC occurred at the highest temperature (33°C). However, in that study the participants were exercising vigorously under high humidity. Gibbons and Adams (1984), at the same concentration of ozone (0.3 ppm) used in this study, reported a non-statistically significant trend towards an ozone–temperature interaction in regards to FEV_1_ and FVC. Epidemiology studies have equally been nondefinitive with few studies showing an interaction of temperature and ozone for lung function; those that have appear to indicate that elevated temperature may result in improved children’s respiratory health perhaps because of behavioral modifications (Li et al. 2012).

This study has several limitations. Although the order in which participants received ozone or air was randomized, as a safety precaution exposures at lower temperatures always preceded those at higher temperatures. It is possible that there is an order effect although this has not been noted in previous studies and ozone effects do not persist after 48 hr (McDonnell et al. 1997). We adjusted for the period within the constraints of the experimental design by using a DOS factor, which was possible because of variation between participants for the length of time between exposures; although this DOS factor does not eliminate the period effect, we believe that it improves the model. However, now that we have completed this study without adverse events, future studies should randomize temperature. Again, for safety reasons, young healthy participants were chosen as the study population. However, epidemiologic studies suggest that the vulnerable subpopulations to heat-related mortality are those with specific cardiovascular diseases, children, and the elderly (Basu 2009). Similarly, studies suggest that our population is at little or no risk to the cardiovascular effects of air pollutants (Pope 2000). The level of ozone used here (0.3 ppm) is comparable with that used in many previous controlled human exposure studies. Nevertheless, improvements in air quality over the last decades means that this level is no longer reached in cities in the United States, although peak hourly concentrations in heavily polluted cities such as Beijing can approximate them (Xu et al. 2008). The amount of ozone that our participants received in 2 hr is equivalent to 8 hr at the current U.S. EPA NAAQS (National Ambient Air Quality Standards) 8-hr ozone standard of 0.076 ppm (U.S. Environmental Protection Agency 2008). Future studies will be performed at lower levels to confirm there is no high-dose effect.

Exposures at elevated temperatures were not performed when the previous day’s mean ambient temperature was > 24°C. No acclimatization was therefore allowed, to approximate the conditions during a heat wave. Several studies have shown that morbidity and mortality are maximal when the temperature is 5°C above the mean for 3 consecutive days (Gasparrini and Armstrong 2011). Our study design does not mimic this scenario, and the responses we found are likely more muted that those in the real world. The aim of these studies however, is not to induce clinical effects but rather to observe physiological effects that are benign and transient in our study individuals, but when extrapolated to the population level could have clinical consequences in susceptible groups such as those with cardiac or respiratory diseases.

In conclusion, our results suggest that ozone at moderate temperatures activates fibrinolysis, whereas at elevated temperature it may impair this pathway. Extrapolated to the population level, this reduction in the efficiency of preventing clot formation and clearance may represent a risk in certain susceptible individuals such as those at risk for thrombosis. These results provide biological plausibility for increased risk from ozone-induced mortality at high temperatures.
